# Macrophage polarization induces endothelium-to-myofibroblast transition in chronic allograft dysfunction

**DOI:** 10.1080/0886022X.2023.2220418

**Published:** 2023-06-08

**Authors:** Zeping Gui, Xiang Zhang, Qianguang Han, Zhou Hang, Ruoyun Tan, Min Gu, Zijie Wang

**Affiliations:** a Department of Urology, The Second Affiliated Hospital of Nanjing Medical University, Nanjing, P.R. China; b Department of Urology, The Affiliated Hospital of Nantong University, Nantong, P.R. China; c Department of Urology, The First Affiliated Hospital of Nanjing Medical University, Nanjing, P.R. China

**Keywords:** Macrophages, polarization, endothelium-to-myofibroblast transition, chronic allograft dysfunction, TNF-α

## Abstract

Our research explores the role of M1 macrophage polarization in endothelium-to-myofibroblast transition (EndMT) and chronic allograft dysfunction (CAD). GSE21374 transcriptome sequencing data were obtained. Transplanted nephrectomy specimens from CAD patients were collected and studied to explore the infiltration of M1 and M2 macrophages using immunofluorescence, PCR, and Western blotting (WB). A co-culture model of M1 macrophages, polarized from mouse bone marrow-derived macrophages (BMDM) or Raw264.7, and aortic endothelial cells was established, and EndMT was tested using PCR and WB. RNA-sequencing was performed on the macrophages from the mouse BMDM. The TNF-α secreted from the polarized M1 macrophages was verified using ELISA. Based on the GEO public database, it was observed that macrophages were significantly infiltrated in CAD allograft tissues, with CD68(+) iNOS(+) M1 macrophages significantly infiltrating the glomeruli of allograft tissues, and CD68(+)CD206(+) M2 macrophages notably infiltrating the allograft interstitial area. The mRNA expression of the M1 macrophage marker inducible nitric oxide synthase (iNOS) was significantly increased (*p* < 0.05) and M1 macrophages were found to significantly promote the EndMT process *in vitro*. RNA-Sequencing analysis revealed that TNF signaling could be involved in the EndMT induced by M1 macrophages, and *in vitro* studies confirmed that TNF-α in the supernatant was significantly higher. The renal allograft tissues of CAD patients were found to be significantly infiltrated by M1 macrophages and could promote the progression of CAD by secreting the cytokine TNF-α to induce EndMT in endothelial cells.

## 1. Introduction

The emergence of human leukocyte antigen (HLA) matching and new immunosuppressive agents dramatically reduced the occurrence of acute rejection after kidney transplantation, while chronic allograft dysfunction (CAD), histologically characterized as renal interstitial fibrosis, continues to account for long-term allograft loss [1]. Endothelial-to-myofibroblast transition (EndMT) characteristically involves endothelial cells losing endothelial cells under various external stimuli, transforming into myofibroblasts, secreting a large amount of extracellular matrix, and promoting fibrosis in the diseased tissues [2]. Our previous studies have reported that endothelial cells are one of the important sources of myofibroblasts, contributing to the pathogenesis of chronic renal interstitial fibrosis and CAD through classical and non-classical signaling pathways [1,3]. Interestingly, it has been demonstrated that various pro-inflammatory cytokines, including tumor necrosis factor-α (TNF-α), Interleukin-2 (IL-2), and transforming growth factor-β1 (TGF-β1), could induce endothelial cells into myofibroblasts in renal diseases [4]. Nevertheless, the origins of these pro-inflammatory cytokines remain unclear under fibrotic conditions.

In recent years, macrophages have been identified as being crucial to the acute phase of rejection and long-term fibrotic formation following renal transplant [5–7]. It is widely recognized that macrophages can be polarized into two different macrophage phenotypes under different stimulus conditions, the classically activated M1 macrophages and the alternative proliferation-activated M2 macrophages [8]. Studies indicate that macrophage polarization could be involved in mediating chronic kidney transplantation damage [9]. In a rat kidney transplant chronic rejection model, the cytokines interleukin-6 (IL-6), monocyte chemoattractant protein-1 (MCP-1), and inducible nitric oxide synthase (iNOS), secreted during macrophage polarization, were also found to be involved in the development of CAD and contributing to chronic transplant kidney injury [10]. In addition, our previous study based on the bioinformatics analysis of CAD samples indicates that macrophage polarization could be significantly associated with the development of renal interstitial fibrosis and CAD, the precise mechanism of which remained to be determined [6].

Therefore, this study was designed to explore the role of macrophage polarization in renal specimens from CAD patients, and subsequently, investigate the effect and mechanism of macrophage polarization on the endothelial cells.

## 2. Materials and methods

### 2.1. Ethics approval and consent to participate

The collection of transplanted kidney tissues from patients with normal renal function and kidney tissue specimens from CAD patients were approved by the Ethics Committee of the First Affiliated Hospital of Nanjing Medical University, and the protocol followed in this study was 2016SR-029, with written informed consent being obtained from all transplant recipients.

### 2.2. Patient selection and specimen collection

All kidney tissues with normal transplant function (control group) were derived from transplant donors who underwent allogeneic kidney transplantation at the First Affiliated Hospital of Nanjing Medical University between January 2015 and January 2020 and displayed stable kidney function after the operation. The puncture specimens of the kidney and the cases of CAD transplanted kidney tissue (CAD group) were all obtained from patients with renal transplantation insufficiency who were followed up in the First Affiliated Hospital of Nanjing Medical University between January 2015 and July 2020.

Inclusion criteria for patients with renal allograft insufficiency: 1. Serum creatinine slowly rises, no obvious symptoms of acute rejection during the progression of the disease, and serum creatinine is greater than 200 μmol/L; 2. The transplanted kidney is insufficient and drug intervention is ineffective; 3. No obvious symptoms of infection are displayed during the progression of the disease.

The pathological tissue specimens were obtained from CAD patients who had undergone donor nephrectomy for secondary allograft transplantation or from CAD kidney punctures due to elevated creatinine and lesions in the transplanted kidney. Renal specimens were fixed with paraformaldehyde, embedded, and sectioned for staining experiments. Some tissues were stored in an RNAkeeper (R501-01, Nanjing Novozymes Company) at −80 °C. Detailed basic demographic data was presented in our previous study [4].

### 2.3. Cells and reagents

Mouse-derived macrophage Raw264.7 was purchased from Shanghai Yuanzhixin Biotechnology Co (Shanghai, China). Mouse bone marrow-derived macrophages were primarily extracted from wild-type Balb/c mice, and the methods have been detailed previously [5]. Mouse aortic endothelial cells (MAECs) were purchased from Otwo Biotech Co (Shenzhen, China). CD68 antibodies (ab125212), inducible nitric oxide synthase (iNOS) antibodies (ab210823), CD31 antibodies (ab24590), α-SMA antibodies (ab21027), Collagen-I antibodies (ab260043), VE-Cadherin antibodies (ab33168), GADPH antibodies (ab9485), Alexa Fluor® 488-labeled donkey anti-mouse secondary antibody (ab150109), Alexa Fluor® 555-labeled donkey anti-rabbit secondary antibody (ab150062) were purchased from Abcam Co (Cambridge, MA, USA); Vimentin antibodies (10366-1-AP), F4/80 antibodies (28463-1-AP), CD206 antibodies (60143-1-Ig), Interferon-γ (IFN-γ) were purchased from Proteintech Co (Chicago, IL, USA); CD206 Cell Flow antibodies (17-2061-80), lipopolysaccharide (LPS; 00⁃4976⁃93) were purchased from Thermo Fisher Scientific Co (Waltham, MA, USA); iNOS Flow Cytology antibodies (130-116-421) was purchased from Miltenyi Biotec Co (Bergisch Gladbach, Germany); The primers were designed and synthesized by Nanjing DynaScience Biotechnology Co. TNF-α Antagonist III (R-7050; 303997-35-5) was purchased from Selleck. Co (Shanghai, China).

### 2.4. Access to public data

The GSE25902 dataset used in this study was obtained from the GEO (Gene Expression Omnibus) public database (www. ncbi. nlm.nih.gov/gds). It contains transcriptome information of post-transplantation punctured graft kidney specimens, dividing the samples into CAD and No-CAD groups for subsequent analysis based on transcriptional information.

### 2.5. Immunofluorescence staining

Paraffin sections were dewaxed in xylene, then rehydrated with gradients of anhydrous ethanol, 95% ethanol, 85% ethanol, and 75% ethanol, placed in a solution of sodium citrate at a concentration of 0.01 mol/L, pH 6.0, boiled for 15 min, and then slowly cooled to room temperature. Later, 3% H2O2 was added and incubated at room temperature for 15 min and washed 3 times with TBST for 5 min each. Immunofluorescence blocking solution was used to close the wash after completion. After blocking, the tissues were incubated overnight at 4 °C in the refrigerator with primary antibodies (CD68 antibody diluted 1:800, iNOS antibody diluted 1:1,000, CD206 antibody diluted 1:200), washed 3 times with TBST for 5 min each, and then mixed with the Alexa Fluor® 488-labeled donkey anti-mouse secondary antibody and the Alexa Fluor 555-labeled donkey anti-rabbit secondary antibody. The tissues were incubated at room temperature for 1 h, washed three times with TBST for 5 min each, incubated with DAPI for 15 min, washed three times with TBST for 5 min each, dripped with an anti-fluorescence quencher, sealed with coverslips, and observed under a fluorescence microscope. 555-labeled proteins are green under fluoroscopy, while Alexa Fluor 555-labeled proteins are red under fluoroscopy.

### 2.6. HE and Masson staining

HE-stained slides were similarly dewaxed and rehydrated as in the immunofluorescence staining process. The tissue slides were stained in hematoxylin solution for 5 min, rinsed with water for 5 min, acidified in 1% hydrochloric acid ethanol for 30 s, rinsed with water for 30 s, eosin solution for 3 min, rinsed with water for 5 min, dehydrated in 75% ethanol, 85% ethanol, 95% ethanol, anhydrous ethanol, placed in xylene, and finally sealed with neutral resin. The Masson stained slides were dewaxed, rehydrated, stained with hematoxylin, and acidified as in the HE staining process; then they were treated with Masson Lixin Red acidic reddish solution for 5-10 min, washed with 2% glacial acetic acid aqueous solution for a few moments, fractionated with 1% phosphomolybdic acid aqueous solution for 3-5 min and stained directly with aniline blue for 5 min, washed with 0.2% glacial acetic acid aqueous solution for a few moments, and finally sealed through dehydration as in the HE staining process.

### 2.7. Macrophages polarization and cell co-culture

To polarize cells toward M1-type macrophages, Raw264.7 cells or mouse BMDMs were induced with 5 ng/mL LPS and 40 ng/mL IFN-γ for 24 h. The polarization efficiency was verified using PCR, cellular immunofluorescence, and flow cytometry. 2 mL of polarized M1 macrophages were placed in the upper layer of 0.4 μm Transwell at 1 × 10^5^ cells/mL, while mouse aortic endothelial cells were planted in the lower layer of Transwell at an inoculation density of 1 × 10^5^ cells/well with DMEM + 10% FBS. After co-culturing for 72 h, total protein and RNA from the lower endothelial cells were extracted for use.

Mouse aortic endothelial cells (MAECs) were grown in the lower layer of the chambers, and Transwell chambers with polarized M1 macrophages were added to the wells of the 6-well plate after 0, 24, and 48 h, respectively, to obtain endothelial cells at 0, 24, 48, and 72 h of M1 macrophage action.

The RAW264.7 macrophage line was selected and polarized toward M2-type macrophages using 40 ng/mL of interleukin (IL)-4 induced macrophage line Raw264.7 for 24 h. The polarization efficiency was verified using PCR, cellular immunofluorescence, and flow cytometry. The co-culture method was employed as before, with the above experiments being repeated thrice.

### 2.8. Polymerase chain reaction

The RNA of all samples was synthesized using HiScript II. qRT-PCR was performed on an AB7300 real-time fluorescence quantification instrument (Applied Biosystems, USA), and the PCR reaction was performed using AA341, a real-time quantitative PCR reagent, while GADPH was used as an internal reference to detect mRNA expression. The process was repeated thrice with each sample, and the data were analyzed by comparing Ct values. As described previously, the specific primers used in this study were bought from Tsingke Biotechnology (Nanjing, China) [5].

### 2.9. Western blot

Once the samples were lysed and the proteins extracted using a RIPA reagent, concentrations were determined and the proteins were separated by 10% SDS⁃PAGE and transferred to polyvinylidene difluoride (PVDF) membranes. After blocking, the strips were placed in primary antibodies (Vimentin, 1:10,000; Collagen I, 1:1,000; GADPH, 1:5,000; VE-Cadherin, 1:1,000; α-SMA, 1:20,000) and incubated overnight at 4 °C. Next, they were TBST washed three times for 15 min each, after which the strips were incubated with secondary antibodies (HRP-labeled anti-mouse IgG, 1:1,000) for 15 min each. The signal was detected with a chemiluminescence system (Bio-Rad, USA) and analyzed using Image Lab software. This experiment was repeated thrice.

### 2.10. Enzyme-linked immunosorbent assay

The enzyme-linked immunosorbent assay (ELISA) kit used in the samples came from the co-culture supernatant of M1-type macrophages, and endothelial cells were removed and centrifuged at room temperature. The relative levels of TNF-α in the supernatants of different co-cultures were measured using an enzyme-linked immunosorbent assay.

### 2.11. Transwell assay

Detailed Transwell assay procedures were presented previously [1]. Briefly, M1-type macrophages or M2-type macrophages were placed in the upper chamber, while endothelial cells were placed in the lower chamber. After 48 h incubation at 37 °C, cell migration was quantified by counting the number of cells present on the lower surface using a phase contrast microscope (Eclipse TS100, Nikon, Japan) at 100× magnification. The Migration Index was calculated according to the following formula (migration index = migration cell number [48 h]/migration cell number [0 h]). The assay was repeated at least three times independently.

### 2.12. RNA-Sequencing and data analysis

RNA integrity was assessed using the RNA Nano 6000 Assay Kit of the Bioanalyzer 2100 system (Agilent Technologies, CA, USA). Total RNA was used as input material for the RNA sample preparations. The detailed procedures were described previously in a published paper [5]. We performed clustering of the index-coded samples on the Cluster Generation System using TruSeq PE Cluster Kit v3-cBot-HS (Illumia) as per the manufacturer’s instructions.

Raw data (raw reads) of fastq format were first processed through in-house Perl scripts. An index of the reference genome was built and paired-end clean reads were aligned. The reads numbers mapped to each gene were counted using FeatureCounts v1.5.0-p3. Subsequently, the FPKM of each gene was calculated based on the length of the gene and the read count mapped to it. Differential expression genes (DEGs) analysis of M1 and M0 groups was performed using the DESeq2 R package (1.20.0). The resulting *P*-values were adjusted using Benjamini and Hochberg’s approach. Genes with an adjusted *P*-value less than 0.05 were considered differentially expressed. The cluster profile R package was used to test the statistical enrichment of differential expression genes in KEGG pathways.

### 2.13. Statistical analysis

We employed R3.6.1 to analyze all data, using the Limma package to differentially analyze public transcriptome data, and the Clusterprofiler package to analyze gene enrichment based on GO (Gene Ontology) and KEGG (Kyoto Encyclopedia of Genes and Genomes) databases. Each sample’s immune cell infiltration status was also fitted (number of fits = 100) using the bioinformatics software Cibersort (cibersortx.stanford.edu). Immunofluorescence intensity and immunohistochemical positive regions were calculated using the ImageJ software. All statistical plots were drawn and counted with the GraphPad software (7.0.1). The Student’s method was used to compare differences between the two groups, with ANOVA being used to compare the measurement data between multiple groups and Tukey’s multiple comparison test also being applied for comparison between the two groups. *p* < 0.05 was considered a statistically significant difference.

## 3. Result

### 3.1. Significant mononuclear macrophage infiltration in renal allograft of CAD patients

**Figure 1.**
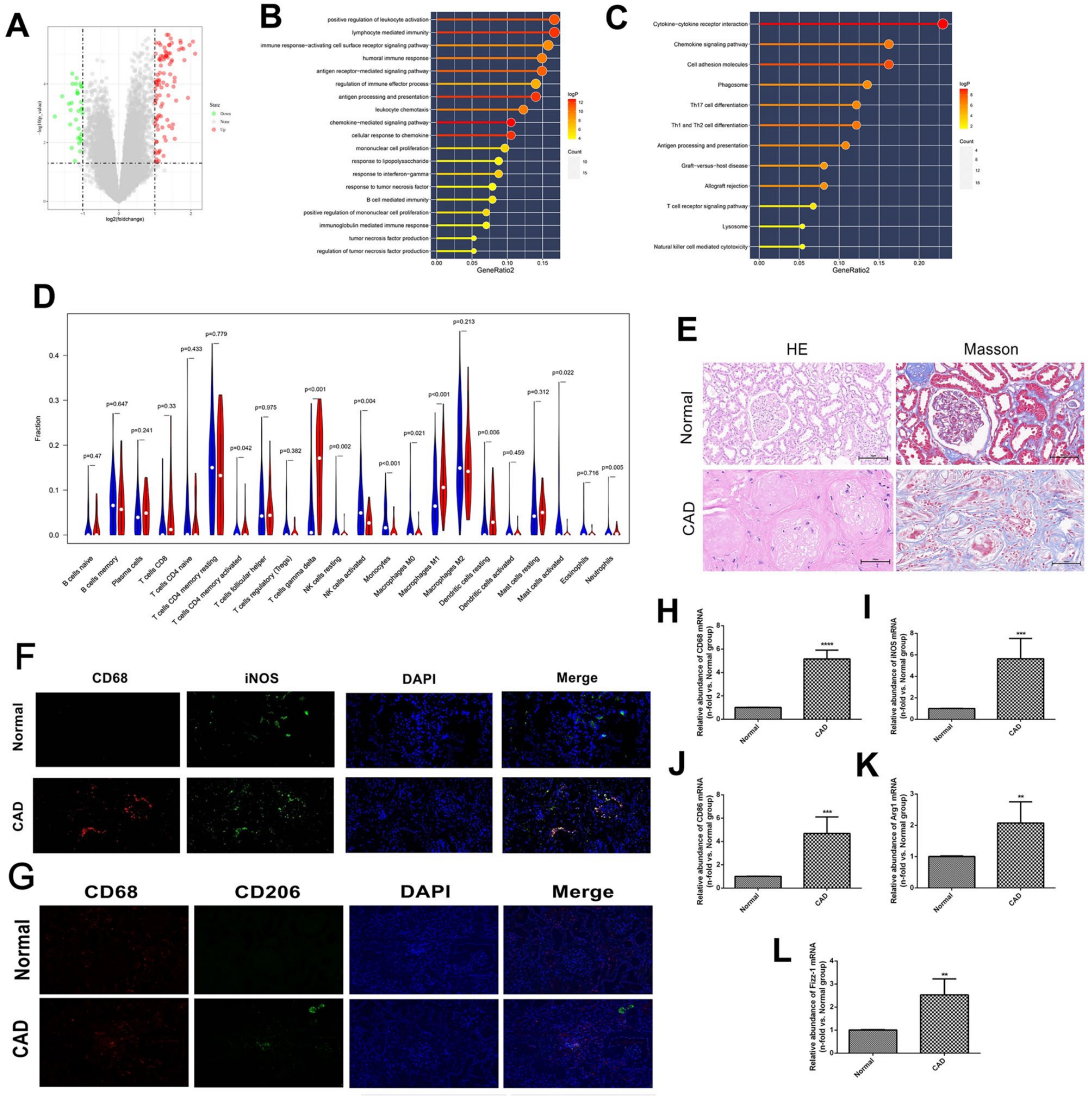
Identification and validation of macrophage polarization in chronic allograft fibrosis. (A) volcano plot of differentiated expressed genes (DGEs) in GSE25902; (B-C) Gene set enrichment analysis of DEGs in GSE25902 in GO (B) and KEGG (C) databases; (D) immune cell infiltration results of DEGs using Cibersort software. The red bars represent the relative abundance of specific immune cells in the CAD group, and the blue bars represent the relative abundance of corresponding immune cells in the stable group; (E) HE and Masson staining results in renal specimens from healthy volunteers (*n* = 5) and patients with chronic allograft dysfunction (CAD; *n* = 5); (F-G) Indirect double immunofluorescence staining of CD68(+) iNOS(+) M1 macrophages (F) and CD68(+) CD206(+) M2 macrophages (G) in Normal (*n* = 5) and CAD (*n* = 5) groups; (H-L) PCR analysis of CD68 mRNA (H), iNOS mRNA (I), CD86 mRNA (J), Arg-1 mRNA (K), and Fizz1 mRNA (L) in Normal (*n* = 5) and CAD (*n* = 5) groups. **p < 0.01 compared to Normal group; ***p < 0.001 compared to Normal group; ****p < 0.0001 compared to Normal group. Each experiment was duplicated at least thrice.

Firstly, the GSE25902 dataset was downloaded and homogenized, and differential analysis of transcriptome information between patients with stable allograft function and those with CAD revealed the presence of various differentially expressed genes (DEGs; Figure 1(A)), which were further analyzed using pathway enrichment analysis based on the GO and KEGG databases. It is indicated that DEGs were mainly enriched in lymphocyte-mediated immunity, positive regulation of leukocyte activation, immune response-activating cell surface receptor signaling pathway, antigen receptor-mediated signaling pathway, and humoral immune response as suggested in the GO database (Figure 1(B)), while in the KEGG database, DEGs were mainly enriched in Cytokine-cytokine receptor interaction, Cell adhesion molecules, Th1 and Th2 cell differentiation, Chemokine signaling pathway, and Antigen processing and presentation (Figure 1(C)). We explored immune cell infiltration in CAD allograft tissue using the Cibersort algorithm, and observed significant monocyte and macrophage infiltration, including Monocytes, M0, M1, and M2 macrophages, in the CAD group (Figure 1(D)).

HE staining disclosed significant glomerulosclerosis, tubular atrophy, and interstitial fibrosis in the CAD group; Masson staining suggested massive collagen fibrin deposition in the interstitium area (Figure 1(E)). Double immunofluorescence assay further identified the significantly higher infiltration of CD68 (+) iNOS (+) M1 macrophages in the glomeruli area of the CAD group (Figure 1(F)), whereas CD68 (+) CD206 (+) M2 macrophages were primarily infiltrated in the interstitial area of the CAD group (Figure 1(G)). Additionally, the expression of CD68 mRNA, a specific indicator of macrophages, was also significantly higher in the CAD group than in the control group (*p* < 0.05, Figure 1(H)). Moreover, the expression of iNOS and CD86 mRNA, specific indicators of M1-type macrophages, were significantly higher in the CAD group (*p* < 0.05, Figure 1(I and J)). Similarly, Arg1 and Fizz-1 mRNA was notably increased in the CAD group when compared to the control group (*p* < 0.05, Figure 1(K and L)).

### 3.2. M1 macrophage polarization induces EndMT process

**Figure 2.**
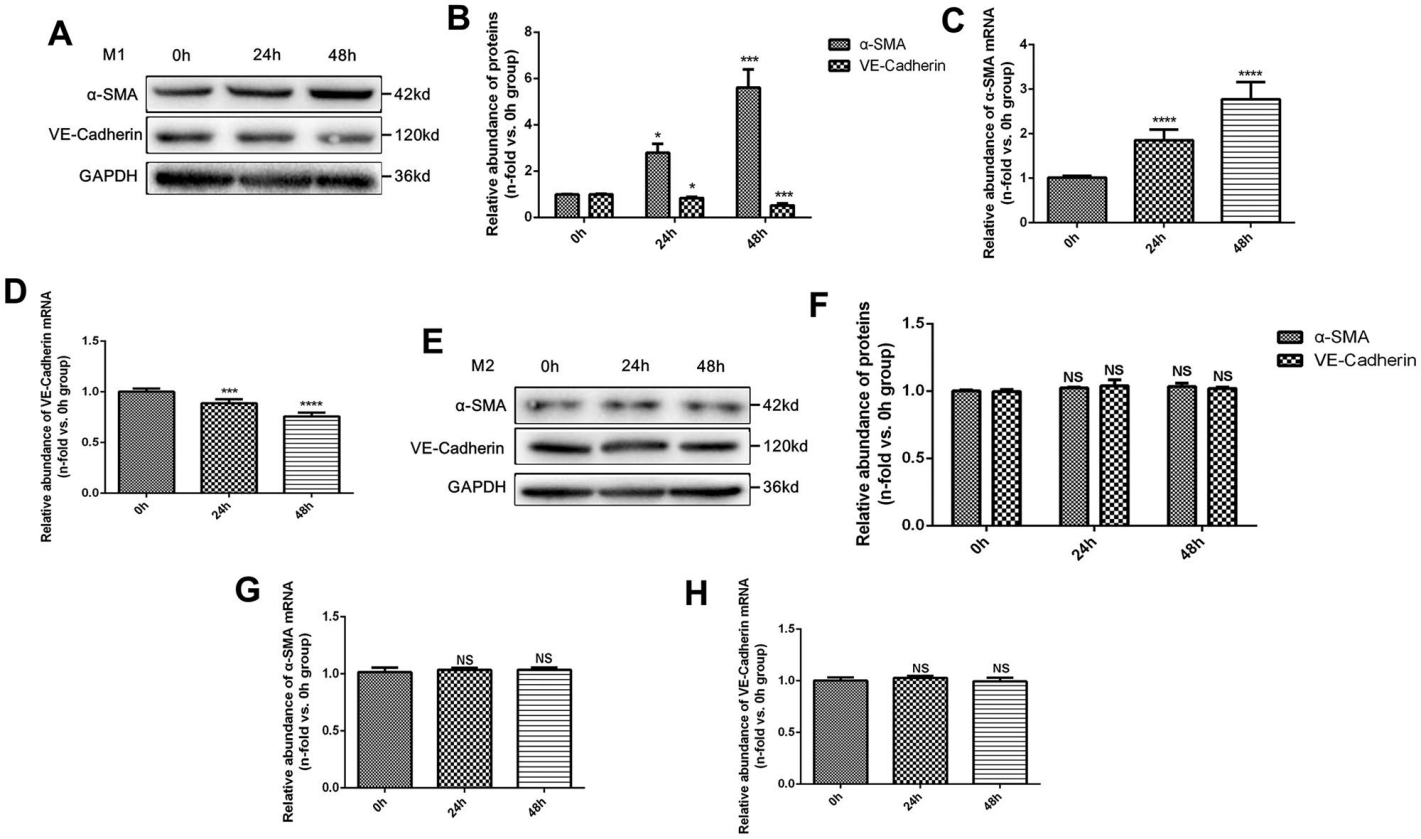
Effects of M1 and M2 macrophages on endothelial cells *in vitro*. (A) Western Blot (WB) results of α-SMA and VE-Cadherin proteins in MAECs treated with M1 macrophages for 0, 24, and 48 h; (B) quantitative analysis of WB results; (C-D) PCR analysis of α-SMA mRNA (C), and VE-Cadherin mRNA (D); (E) WB results of α-SMA and VE-Cadherin proteins in MAECs treated with M2 macrophages for 0, 24 and 48 h; (F) quantitative analysis of WB results; (G-H) PCR analysis of α-SMA mRNA (G), and VE-Cadherin mRNA (H). *p < 0.05 compared to 0h group; ***p < 0.001 compared to 0h group; ****p < 0.0001 compared to 0h group; NS, no significance compared to 0h group. Each experiment was duplicated at least thrice.

M1 and M2 macrophages were polarized *in vitro* and co-cultured with MAECs, respectively. The results indicated that the expression of α-smooth muscle actin (α-SMA) protein was significantly higher when induced by M1 macrophages in a time-dependent manner, while the expression of a VE-Cadherin protein, a specific marker of endothelial cells, remarkably decreased after co-culture (Figure 2(A and B)). The PCR results of the increased expression of α-SMA mRNA and the decreased expression of VE-Cadherin mRNA were similar to a protein assay (Figure 2(C and D). In contrast, no significant difference between α-SMA and VE-Cadherin proteins was observed when MAECs were co-cultured with M2 macrophages (Figure 2 (E and F)) and when MAECs were treated with M2 macrophages (Figure 2(G and H)).

**Figure 3.**
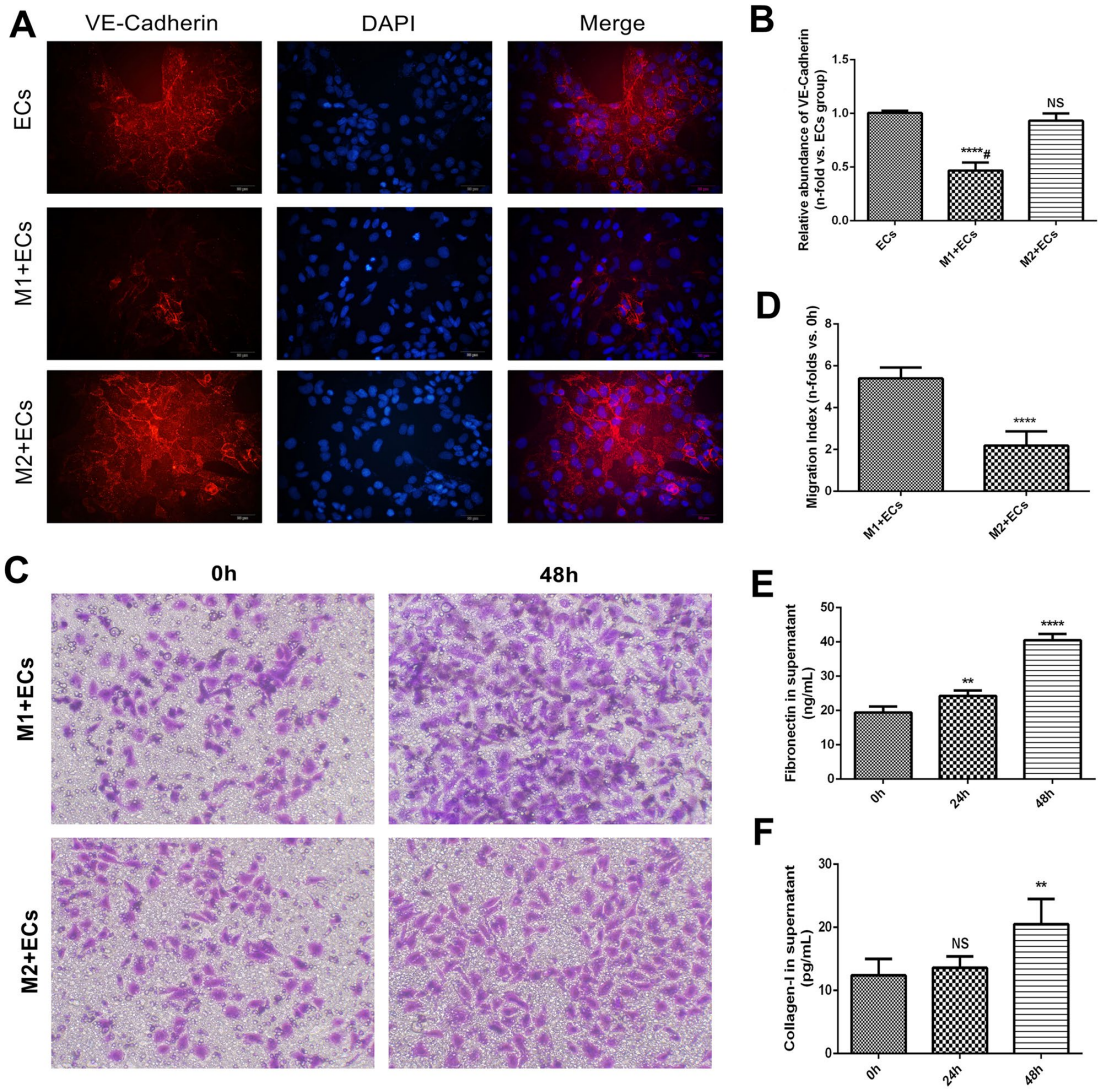
Functional examination of MAECs treated with macrophages. (A) Indirect immunofluorescence staining of VE-Cadherin in MAECs treated with M1 and M2 macrophages; (B) relative abundance of VE-Cadherin immunofluorescence staining among three groups (****p < 0.0001 compared to ECs group; NS, no significance compared to ECs group; ^#^*p* < 0.0001 compared among three groups); (C) Transwell assays of MAECs treated with M1 and M2 macrophages; (D) Migration index of MAECs in two groups (****p < 0.0001 compared to M1 + ECs group); (E-F) concentrations of Fibronectin (E) and Collagen-I (F) in the supernatant of MAECs treated with M1 and M2 macrophages (**p < 0.01 compared to 0h group; ****p < 0.0001 compared to 0h group; NS, no significance compared to 0h group). Each experiment was duplicated at least thrice.

Then, a cell immunofluorescence assay was used to compare the distribution and expression of VE-Cadherin protein in endothelial cells. We found that VE-Cadherin protein significantly decreased in endothelial cells treated with M1 macrophages when compared to cells co-cultured with M2 macrophages (Figure 3(A and B)). Moreover, transwell assays revealed a dramatic increase in the migration ability of endothelial cells treated with M1 macrophages (Figure 3(C and D)). In addition, Collagen-I and Fibronectin in the supernatant of endothelial cells treated with M1 macrophages were also elevated (Figure 3(E and F)).

### 3.3. RNA-Sequencing analysis suggested the involvement of a TNF signaling pathway in EndMT induced by M1 macrophage

**Figure 4.**
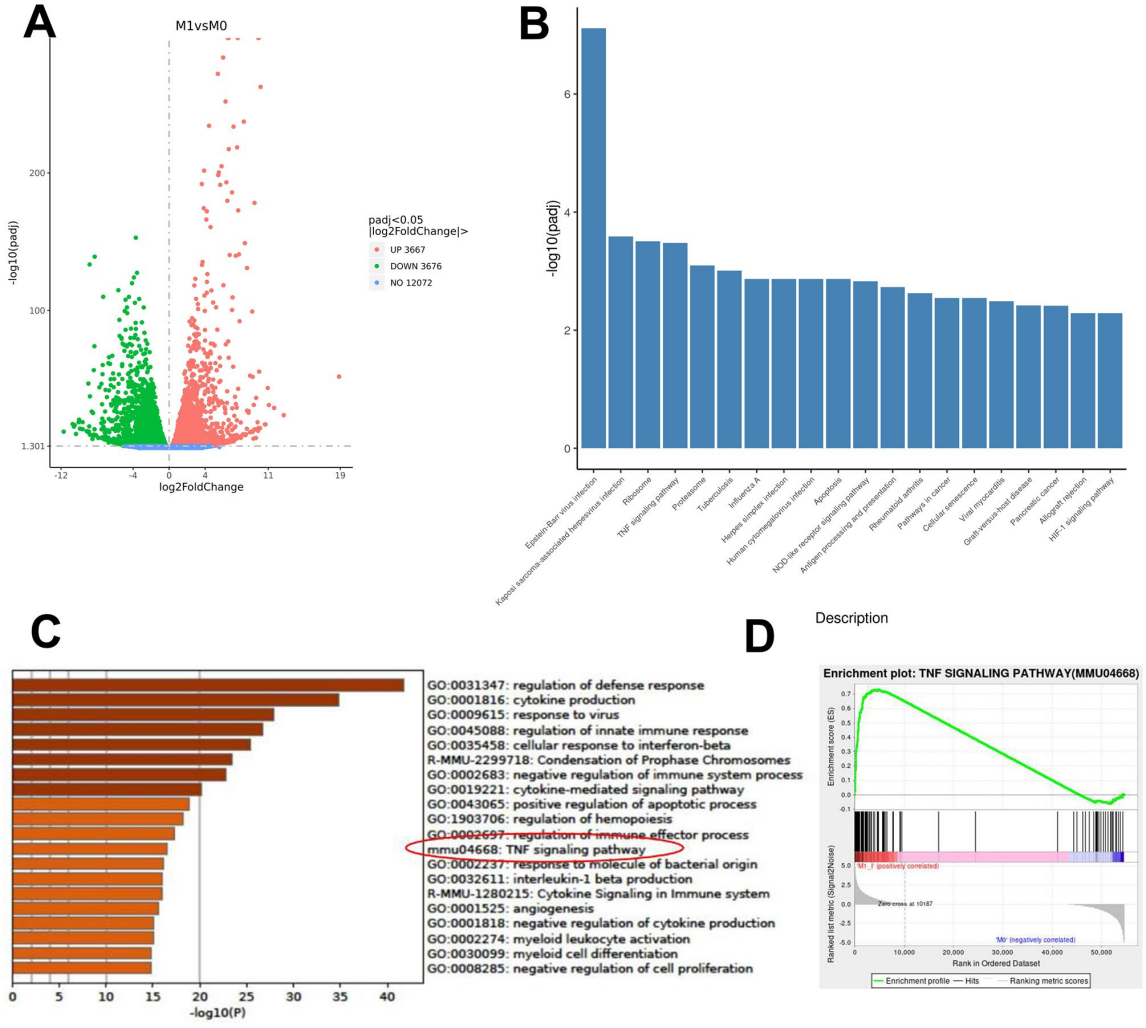
RNA sequencing and data analysis of M1 macrophages. (A) Volcano plot of differentiated expressed genes (DGEs) between M1 and M0 macrophages; (B) Gene set enrichment analysis of DEGs in KEGG databases; (C) data on TNF signaling pathway graph in KEGG databases; (D-E) gene set enrichment analysis of DEGs in GSE25902 in GO databases.

To further explore the potential mechanism involved in EndMT induced by M1 macrophages, we performed RNA-Sequencing using M0 and M1 macrophages, ultimately identifying a total of 7343 DEGs (3667 upregulated and 3676 downregulated), for further functional analysis (Figure 4(A); Supplemental Table 1). We then carried out a KEGG/GSEA functional enrichment analysis of DEGs between M1 and M0, enriching significant biological functions including Epstein-Barr virus infection, Kaposi sarcoma-associated herpesvirus infection, Ribosome, TNF signaling pathway, and Proteasome (Figure 4(B)). Consequently, the TNF signaling pathway in KEGG was selected for extensive analysis using the Pathview software. As illustrated in Supplemental Figure 1, TNF-related proinflammatory cytokines were significantly elevated, while the PI3K-Akt signaling pathway in macrophages was down-regulated and induced by TNF signaling. In addition, the functional enrichment analysis in this study was validated by employing the GSE25902 dataset, and the TNF signaling pathway was also expressly noted (Figure 4(C and D)).

### 3.4. M1 macrophages induce the EndMT process through the TNF signaling pathway

**Figure 5.**
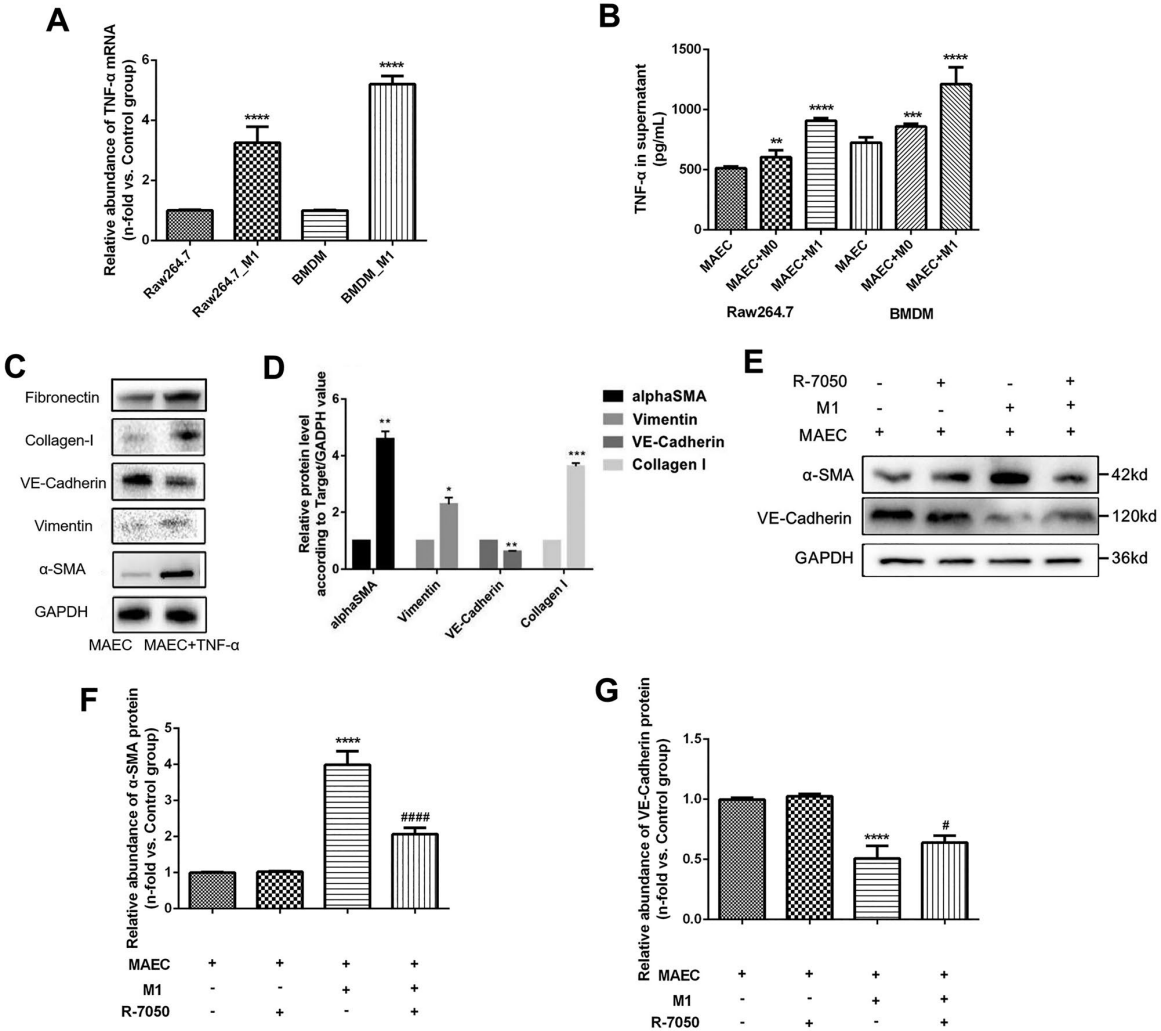
TNF signaling pathway examination in MAECs cells treated with M1 macrophages. (A) Relative abundance of TNF-α mRNA in M1 macrophages by PCR; (B) TNF-α cytokines in the supernatant of MAECs treated with M1 macrophages; (C) WB results of α-SMA, Vimentin, and VE-Cadherin proteins in MAECs treated with TNF-α cytokines; (D) quantitative analysis of WB results; (E) WB results of α-SMA and VE-Cadherin proteins in MAECs treated with M1 macrophages and/or R-7050; (F-G) quantitative analysis of WB results of α-SMA (F) and VE-Cadherin (G) proteins. *p < 0.05 compared to control group; **p < 0.01 compared to control group; ***p < 0.001 compared to control group; ****p < 0.0001 compared to control group; ^#^*p* < 0.05 compared to intervention group with M1 and MAEC; ^####^*p* < 0.0001 compared to intervention group with M1 and MAEC. Each experiment was duplicated at least thrice.

To validate our findings from the RNA sequencing, TNF-α mRNA was examined in both Raw 264.7 cells and BMDMs. Figure 5(A) indicates significantly increased TNF-α mRNA in both cells (*p* < 0.001). Furthermore, concentrations of TNF-α in the supernatant from each group were also tested, and similar results were obtained (Figure 5(B)). Our previous study confirmed the pro-fibrotic effect TNF-α had on endothelial cells [4], which is consistent with our *in vitro* study (Figure 5(C and D)). To further explore the TNF signaling pathway involved in the EndMT induced by M1 macrophages, a specific inhibitor of the TNF-α receptor (R-7050) was applied to MAECs co-cultured with M1 macrophages. Our findings suggest that R-7050 could significantly inhibit the up-expressed α-SMA proteins and increase the expression of VE-Cadherin proteins, indicating that the progression of EndMT induced by M1 macrophages were attenuated by R-7050 *in vitro* (Figure 5(E and G)).

## 4. Discussion

In our study, both M1 and M2 macrophages were observed to significantly infiltrate the allograft tissue of CAD patients both in the GEO public database and in the allograft specimens from our center. *In vitro* experiments revealed that M1 macrophage polarization significantly induced the EndMT process in endothelial cells through the TNF signaling pathway, while M2 macrophages failed to induce the EndMT process.

M1 macrophage polarization significantly infiltrates CAD renal allograft and may contribute to the development of CAD by inducing the EndMT process [1]. Our previous studies have already reported that the renal microvascular EndMT process is significantly activated during the formation of renal allograft interstitial fibrosis, of which tissue growth factor-β1 (TGF-β1) and TNF-α were considered crucial to the induction [1,4]. Nevertheless, the origins of these pro-fibrotic cytokines remained unknown. In this study, M1 macrophage was identified as an important source of TNF-α cytokines in renal allograft fibrosis. Differing from chronic renal fibrosis, renal allograft fibrosis was characterized as a comprehensive biological process induced by both immunological and non-immunological factors, with chronic active antibody-mediated rejection, inadequate immunosuppression, and virus infection tending to contribute to the immunological promotors [11,12]. Interestingly, the application of mesenchymal stromal cells (MSCs) with immunosuppression and regenerative properties presented safety and feasibility results in attenuating the long-term allograft loss, partly attributable to declined acute rejection [13–15]. Consistent with these results, the immunosuppressive agents targeting inherent immunity could offer promising results in renal allograft fibrosis by reducing the infiltration of pro-inflammatory cells and subsequently pro-inflammatory cytokines, such as TNF-α [16,17]. Therefore, our results provide concrete evidence for the application of immunosuppressive agents to mitigate the process of renal allograft fibrosis.

The local immune microenvironment of a solid organ or tissue can contribute to the development and progression of organ-specific diseases [18], and this microenvironment existing in renal allograft contains both specific and inherent immunity. It has been found that activation of renal allograft colonized macrophages is associated with allograft survival [19], while evidence also proves that bone marrow-derived macrophages colonized in the transplanted kidney are capable of transforming into myofibroblasts, leading to the deterioration of chronic allograft failure [20]. The role of polarized macrophages in chronic allograft failure and injury has been evidenced in several previous studies, for instance, the characteristic genes and secretory factors of M1-type macrophages that can lead to chronic allograft injury have been identified and are associated with the long-term prognosis [21]. However, this study also indicated that M2 macrophages, characterized by their wound-healing function, could be significantly infiltrated in chronic allograft fibrosis, which has been confirmed as being crucial in chronic renal fibrosis [22]. Several pro-fibrotic cytokines secreted by M2 macrophages, such as TGF-β1, are regarded as the main mechanisms involved in renal fibrosis, and subsequent pathogenesis remains unclear. In this study, the M2 macrophage failed to induce the EndMT process, suggesting direct effects and mechanisms could form a part of renal allograft fibrosis induced by M2 macrophages, such as myofibroblast transition [23].

Studies have reported that numerous signaling pathways are involved in chronic renal fibrosis and chronic allograft fibrosis, such as the Wnt/β-catenin signaling pathway, phosphatase 2 A signaling pathway, and autophagy [24–26]. Previous studies have focused on cytokines and signaling pathways leading to the phenomenon of EndMT, with fewer studies focused on the sources of EndMT-inducing signaling factors, and a lack of correlated studies on the effects of the local immune microenvironment of the transplanted kidney on EndMT [27]. In this study, TNF signaling was identified as being crucial for the induction of EndMT, indicating that inhibition of the secretion of TNF-α and TNF signaling pathways in M1 macrophages could prove promising for chronic allograft fibrosis. While prior studies focused on the TNF signaling pathway in acute renal injury and renal ischemia-reperfusion injury considering its pro-inflammatory property [28,29], this study’s findings from the bioinformatics analysis and *in vitro* experiments demonstrated the pro-fibrotic effect of TNF signaling in chronic renal allograft fibrosis, offering a novel insight for further exploration.

Indeed, we have noticed several major limitations that impede obtaining strong evidence of M1 macrophage on EndMT. Firstly, TNF signaling needs to be extensively explored using knockout mice and inhibitory plasmids. Besides, mediators between M1 macrophages and endothelial cells are not limited to cytokines, and exosomes secreted by macrophages have to be considered.

## 5. Conclusions

In summary, this study reports significant M1 and M2 macrophage infiltration in both the GEO public database and CAD renal allograft tissues. It confirms that M1 macrophage polarization significantly promotes the EndMT process in endothelial cells through the TNF signaling pathway, while M2 macrophages fail to induce EndMT. Further studies need to be carried out to investigate the extensive mechanisms involved in M1 macrophages and endothelial cells.

## Supplementary Material

Supplemental Material

## Data Availability

The data supporting this study’s findings can be obtained from the corresponding author upon reasonable request.
