# Supplementary figures and images for: Macrophage polarization induces endothelium-to-myofibroblast transition in chronic allograft dysfunction
Source: Ren Fail. 2023 Jun 8;45(1):2220418. doi: 10.1080/0886022X.2023.2220418 (PMC10251776; doi:10.1080/0886022X.2023.2220418)

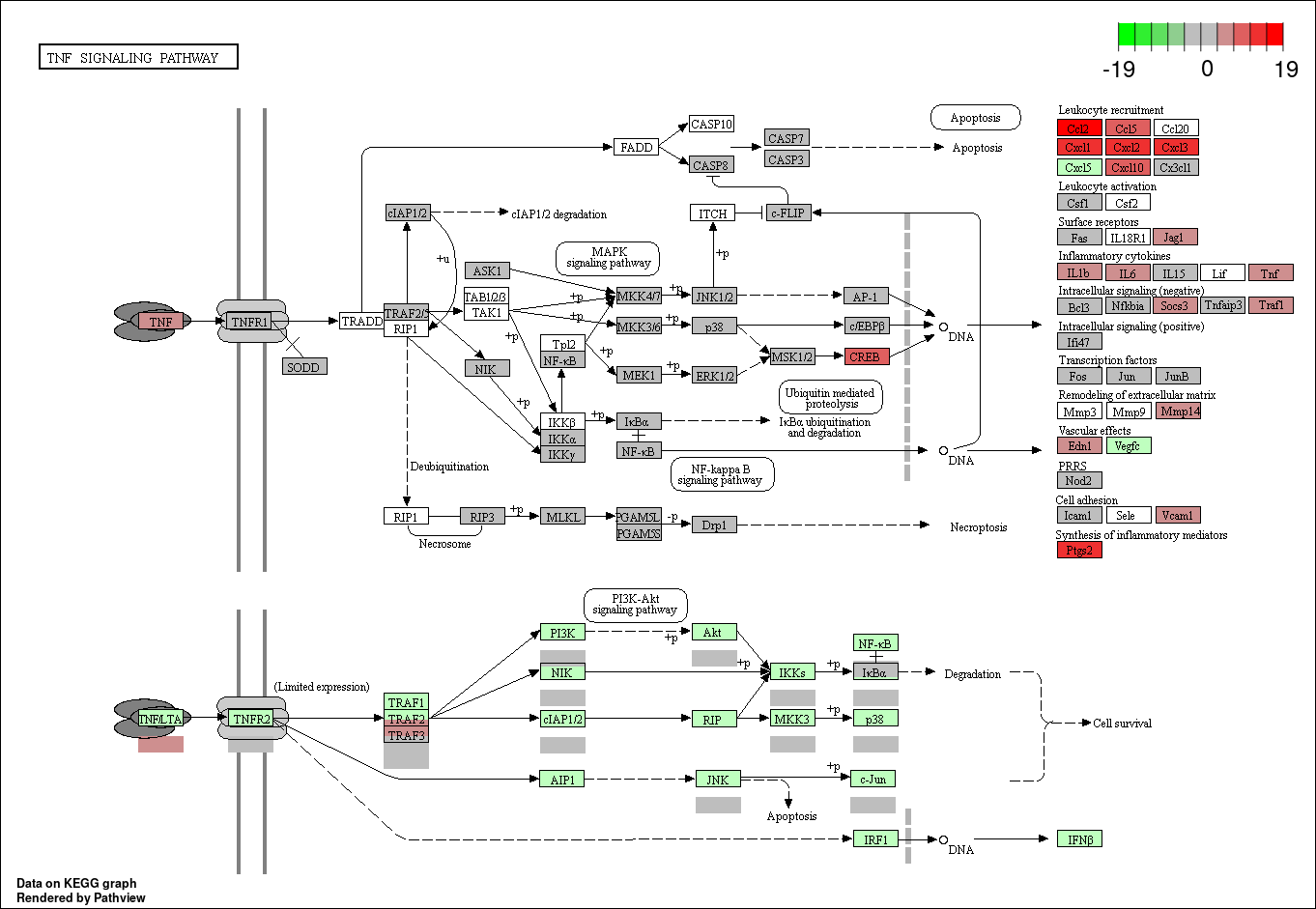

Supplement: Supplemental Material [file IRNF_A_2220418_SM9912.png]
